# Proteomic Insights into Venous Thromboembolism

**DOI:** 10.3390/medsci14010094

**Published:** 2026-02-15

**Authors:** Oana-Mădălina Manole, Brîndușa Alina Petre, Viviana Onofrei

**Affiliations:** 1 Internal Medicine Department, Faculty of Medicine, “Grigore T. Popa” University of Medicine and Pharmacy, 700115 Iasi, Romania; manole.oana-madalina@d.umfiasi.ro (O.-M.M.); onofreiviviana@gmail.com (V.O.); 2 Cardiology Department, “Saint Spiridon” Emergency Clinical County Hospital, 700115 Iasi, Romania; 3 Faculty of Chemistry, Alexandru Ioan Cuza University of Iasi, 11 Carol I Bd., 700506 Iasi, Romania; 4 Center of Fundamental Research and Experimental Development in Translational Medicine (TRANSCEND), Regional Institute of Oncology, General Henri Mathias, No. 2–4, 700483 Iasi, Romania

**Keywords:** venous thromboembolism, proteomics, diagnostic, prognostic

## Abstract

Venous thromboembolism (VTE), including pulmonary embolism (PE) and deep vein thrombosis (DVT), remains a major cause of morbidity and mortality worldwide, with significant clinical challenges in diagnosis and risk stratification. Traditional diagnostic tools, including clinical prediction scores, D-dimer testing, and imaging, are limited by suboptimal specificity or sensitivity. In this context, proteomics-based approaches have emerged as powerful tools to elucidate the molecular mechanisms of VTE and to identify novel diagnostic and prognostic biomarkers. This review synthesizes recent advances in proteomic research relevant to VTE. We searched four databases (PubMed, ScienceDirect, Springer Nature, and Wiley) using the keywords “acute pulmonary embolism”, “acute venous thromboembolism”, and “proteomics”. Thirty proteomic studies investigating VTE were examined. Across these studies, proteomic profiling consistently revealed alterations in pathways related to coagulation, inflammation, platelet activation, endothelial dysfunction, and fibrin clot structure. Multiple protein classes, including acute-phase reactants, complement components, coagulation factors, and platelet-derived proteins, have demonstrated potential value in improving diagnostic accuracy and refining prognostic stratification. Proteomic analyses have also revealed distinct molecular signatures between isolated PE and isolated DVT, supporting the concept of biologically heterogeneous VTE phenotypes. Furthermore, emerging evidence from COVID-19–associated thrombosis, cancer-associated VTE, and non-invasive sources such as exhaled breath condensate underscores the expanding clinical relevance of proteomic approaches. Although technical limitations and heterogeneity across studies remain challenges, the integration of proteomic data with clinical and genetic information holds promise for advancing precision medicine in VTE.

## 1. Introduction

Venous thromboembolism (VTE) is a condition with a significant global epidemiological impact, being the third leading cause of mortality. Pulmonary embolism (PE) and deep vein thrombosis (DVT) are the two phenotypes of VTE. The clinical presentation of acute PE is nonspecific, and the diagnosis is established based on the correlation of clinical suspicion, clinical probability scores (Wells and Geneva), D-dimer levels (age-adjusted), and imaging studies (transthoracic echocardiography and thoracic computed tomography pulmonary angiography (CTPA)) [1]. D-dimer testing has an acceptable sensitivity (83% for point-of-care testing, 94% for enzyme-linked immunosorbent assay (ELISA) but a low specificity (71% for point-of-care testing, 53% for ELISA), as elevated values may also be found in other conditions [2]. The pattern of right heart strain identified on echocardiography has good specificity (80–90%) [3], but limited sensitivity (40–50%) for the diagnosis of acute PE [1,3]. Thoracic CTPA is the gold standard for diagnosing acute PE, with a specificity of 96%. Its main limitation is the subsegmental localization [1]. The short-term severity of acute PE is stratified according to the Pulmonary Embolism Severity Index (PESI) and its simplified version (sPESI), as well as clinical findings (hemodynamic instability), biological markers (cardiac troponin, N-terminal pro–B-type natriuretic peptide, heart-type fatty acid–binding protein, copeptin), and imaging findings [1].

Improving diagnostic and prognostic methods has been a topic of research in recent years. Non-targeted or targeted proteomic profiling has been extensively carried out as a tool to provide new insights into mechanisms for VTE. Some new high-performance diagnostic and prognostic indicators have been obtained through high-resolution liquid chromatography–tandem mass spectrometry (LC–MS/MS), MALDI-TOF/MS, or data-independent acquisition mass spectrometry (DIA-MS) [4]. This research aimed to examine and synthesize recent advances in proteomic analysis with relevance to VTE, focusing on their potential applications in screening strategies, diagnostic pathways, and prognostic stratification. The review addresses both major clinical phenotypes of VTE–PE and DVT—as well as selected specific clinical contexts in which proteomic approaches may offer additional diagnostic or prognostic value.

We searched databases such as PubMed, ScienceDirect, Springer Nature, and Wiley between 12 August and 12 September 2025, to identify studies that performed proteomic analysis in acute PE. The search strategy utilized MESH terms combined with the Boolean operators “AND” and “OR”. The terms included in the search were “acute pulmonary embolism”, “acute venous thromboembolism”, and “proteomics”. Therefore, it was not registered in PROSPERO and no risk analysis was performed, but the search strategy was carried out in accordance with the PRISMA statement [5] to ensure the highest quality.

**Figure 1:**
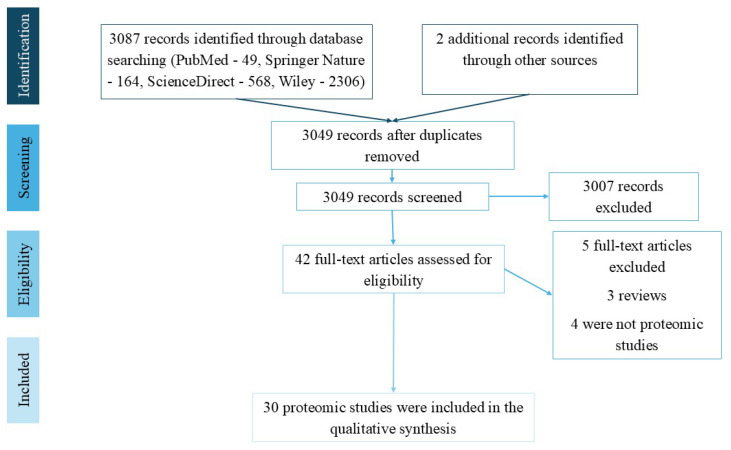
PRISMA flow chart illustrating the study selection process for qualitative synthesis.

The inclusion criteria were also adapted for preclinical studies, as proteomic analysis was investigated in experimental animal models. The inclusion criteria were (1) original reports, (2) containing at least one proteomic analysis, (4) full-text articles, (5) published before September 2025. Two researchers independently reviewed and assessed each extracted study according to the PRISMA guidelines [5] to ensure the highest quality. A total of 3087 records were generated as a result of the search. Two records identified in bibliographic sources were added. Firstly, we found 40 duplicates and removed them. Of the 3049 screened results, 3007 records were excluded. Exclusion criteria were that the article was not suitable (*n* = 2995) or it was an editorial or a conference proceeding article *(n* = 12). A total of 42 full-text articles were assessed for eligibility. After excluding review articles (*n* = 3), and not suitable full-text articles (*n* = 9), a total of 30 proteomic studies met the inclusion criteria and were discussed in this review. Four studies were found that do not use proteomic analysis but provide important information about potential biomarkers in the diagnosis and prognosis of VTE, and these were also discussed. The PRISMA flow chart is illustrated in Figure 1.

## 2. Insights from Recent Studies

Proteomics has emerged as a key tool for understanding the pathophysiology of VTE and for identifying diagnostic, prognostic, and predictive biomarkers, particularly in PE and DVT. Recent studies demonstrate that proteomic alterations reflect the complex interplay between inflammation, coagulation, platelet activation, and endothelial dysfunction. Proteomic analysis has proven effective in increasing the accuracy of PE diagnosis.

Ganesh et al. (2007) compared the serum proteomic profile of 38 patients with VTE (13 patients with isolated DVT, 19 patients with isolated PE, and 6 patients with DVT and PE) and 38 controls, using MALDI-ToF MS and two-dimensional gel electrophoresis (2DGE) as a validation test [6]. This study was the first to apply direct MS to cardiovascular diseases, assessing whether serum proteomic profiles could detect VTE and how their diagnostic performance compared with the conventional D-dimer test. A protein panel including haptoglobin (Hp) and alpha-1B glycoproteins correctly identified 77% of VTE cases and 89% of non-VTE subjects. This predictive model achieved an AUC of 0.85 on ROC curves of sensitivity and specificity, compared with AUC values of 0.70 and 0.62 for D-dimer levels measured by ELISA and immunoturbidimetric methods, respectively. Furthermore, it showed that direct MS is a useful method for assessing VTE, with moderate sensitivity (60%), but increased specificity (80%) compared to other methods such as ELISA, latex agglutination, and immunoturbimetry [6]. Although the study was exploratory and included a relatively small sample size, the findings provide a foundation for identifying potential MS-based biomarkers for VTE. The majority of proteins included in the model were associated with the coagulation cascade or the acute-phase protein family. Although these proteins may constitute promising biomarker candidates for VTE, the presence of comorbidities in the study population, such as trauma, cancer, or surgery, may have acted as potential confounding factors and should be considered when interpreting the findings.

Experimental PE animal models were employed for proteomic analysis in several studies. The PE model in rats was induced by injecting 3–4 emboli into the left jugular vein. The lung tissue protein [7] and serum proteins [8] of the acute PE rat models were identified using matrix-assisted laser desorption/ionization time-of-flight mass spectrometry (MALDI-ToF MS), followed by Western blot analysis [7,8]. Proteomic analysis of the lung tissue samples identified 32 proteins involved in different functions: metabolism of energy, apoptosis and injury, regulation of systemic acid–base balance and blood pressure, associated with the functions of smooth muscle cells or regulation of transcription and translation [7]. Significant modulation of binding proteins such as vitamin D–binding protein (DBP), retinol-binding protein 4 (RBP4), and transthyretin (TTR), alongside acute-phase proteins including Hp and fibronectin (Fn), was observed in serum [8]. DBP is synthesized by the liver and plays an important role in inflammation and injury. It potentiates the chemotactic activity of neutrophil chemoattractants and binds actin released from cellular injury [9]. RBP4, the retinol carrier, possesses proinflammatory effects mediated partially through Toll-like receptor 4 (TLR4) [10]. TTR is a tetrameric thyroid hormone binding protein, involved in reactive oxygen species (ROS) balance [11]. Fn is an extracellular matrix protein that specifically binds other components of the extracellular matrix, signaling molecules, and cell adhesion molecules [12]. Hp binds hemoglobin and is an acute-phase protein [13]. The DBP, RBP4, and TTR serum levels were lower in rats after PE, because the synthesis in the liver was downregulated, while Hp and Fn serum concentrations were increased. The mRNA expression of Hp and Fn was upregulated in the liver and lungs, but mRNA expression of DBP, RBP, and TTR was downregulated in the liver but not in the lungs, because they are expressed under stressful conditions of acute PE in the lungs. These findings were subsequently confirmed in patients with VTE, supporting their biological and clinical relevance. The serum concentration of Hp and fibronectin was higher in patients with VTE than in controls [8]. These results help to understand the involvement of the inflammatory system in the pathophysiology of PE.

### 2.1. Inflammation

Inflammation plays a central role in VTE pathophysiology. As mentioned earlier, Hp is a tetrameric protein belonging to the acute phase protein family. Its main role is to bind hemoglobin with high affinity and prevent endothelial injury during hemolysis [13]. Elevated levels of Hp were found in alveolar macrophages or in inflamed human lung tissue, but not in normal lungs, suggesting its role in hemoglobin clearance and tissue protection against hemoglobin-mediated oxidative stress. Hp is among the most extensively studied acute-phase proteins in PE. Proteomic and clinical studies have reported both increased and decreased circulating levels, depending on disease severity and the presence of hemolysis. These findings underscore the complex interaction between inflammation, pulmonary hypertension, and erythrocyte destruction in acute PE.

In the first study of untargeted proteomic analysis for prognosis in humans acute PE, Hp was identified as a biomarker of severity. Patients with high/intermediate risk acute PE had lower circulating Hp levels than those with low risk (2.6 ± 1.0 g/L vs. 2.3 ± 0.8 g/L, *p* = 0.04). The cutoff value of 1 g/L for Hp has a predictive capacity for high-risk acute PE with a sensitivity of 80% and a specificity of 96%. These results support the strong interaction between the inflammation and thrombosis process [14].

Zhang et al. (2018) conducted a study aimed to identify serum biomarkers for the diagnosis of acute PE, given the limitations of classic markers such as D-dimer [15]. The study design included two phases: a screening phase and a validation phase. In the screening stage, significantly different levels of eight proteins—leucine-rich alpha-2-glycoprotein, Hp, trypsin-3, ITIH4, clusterin, keratin type I cytoskeletal 9, RBP4, and alpha-1-microglobulin/bikunin precursor—were identified in patients with acute PE using 2-DE and MALDI-ToF-MS. In the validation stage, elevated Hp serum levels in PE were confirmed by ELISA. Furthermore, histochemical analysis of lung tissue demonstrated Hp expression in thrombus material, as well as within the intima and media of pulmonary arteries in PE, whereas in controls, it was detected only within the arterial media. Using ROC curve analysis, the researchers used a cutoff value of 256.74 mg/dL for Hp demonstrated diagnostic biomarker potential for acute PE, with a sensitivity of 62% and a specificity of 83% [15]. Table 1 provides a comparative overview of the studies analyzing inflammation in VTE.

PE causes pulmonary hypertension, which is associated with hemolysis. Hemolysis may usually occur through two mechanisms. First, pulmonary hypertension increases the trans-tricuspid pressure gradient, leading to tricuspid regurgitation. This, in turn, causes repeated passage of erythrocytes through the tricuspid valve, ultimately leading to mechanical hemolysis. Second, the elevated pulmonary vascular resistance, where blood flow persists through non-occluded pulmonary arteries and capillaries, generates increased shear stress at the local interface, resulting in red blood cell destruction. An experimental animal model was used to evaluate hemolysis and clearance methods for hemolysis products in cases of PE and/or pulmonary hypertension. Polystyrene microspheres of 25 ± 1 μm were injected intrajugularly in variable amounts of 1.3, 1.65, or 2 million/100 g body weight in Sprague Dawley rats to induce progressive right ventricular systolic pressures. Severe PE, which caused pulmonary vascular resistance >50 mmHg, led to complete clearance of Hp within 2 h and positive modulation of the HMOX1 enzyme [16].

Pentraxin-3, another acute-phase protein with a more significant role in vascular inflammation than protein C reactive (PCR), is a severity indicator in acute PE. Plasma levels of pentraxin-3 were correlated with the risk level of PE—5.83 ± 0.52 ng/mL and 0.93 ± 0.14 ng/mL in high and medium risk, and 0.64 ± 0.13 ng/mL in low risk. Also, elevated values for pentraxin-3 (>3 ng/mL) showed positive correlation with cardiac death and PE recurrence [17].

Complement factor H-related 5 protein (CFHR5), a regulator of the alternative complement pathway associated with C3a-mediated platelet-activation in thrombosis, was correlated with VTE in VEBIOS ER and Coagulation study. The plasma CFHR5 levels were higher in patients with VTE (3430 ± 782 ng/mL) compared with patients without VTE (2840 ± 756 ng/mL). In addition, CFHR5 correlated with unprovoked VTE and recurrence risk [18].

### 2.2. Clot Composition

Proteomic approaches allow differentiation between venous thrombosis and arterial atherothrombosis. Hong et al. (2009) identified four plasma polypeptides with *m*/*z* of 3318, 33,378, 68,125, and 5271 Da capable of distinguishing DVT from acute myocardial infarction with an accuracy of 82.5%, using SELDI-ToF MS [19]. The results highlight distinct molecular mechanisms underlying venous and arterial thrombosis. In addition, polypeptides with *m*/*z* of 5914 Da differentiated the patients with DVT from healthy subjects with a sensitivity and specificity of 100% [19].

The differences between arterial and venous thrombosis were also revealed by proteomic characterization of fibrin clots. These studies have demonstrated that fibrin clots contain a complex network of coagulation factors, inflammatory proteins, complement components, and neutrophil extracellular traps, all of which influence clot structure and susceptibility to fibrinolysis.

**Figure 2:**
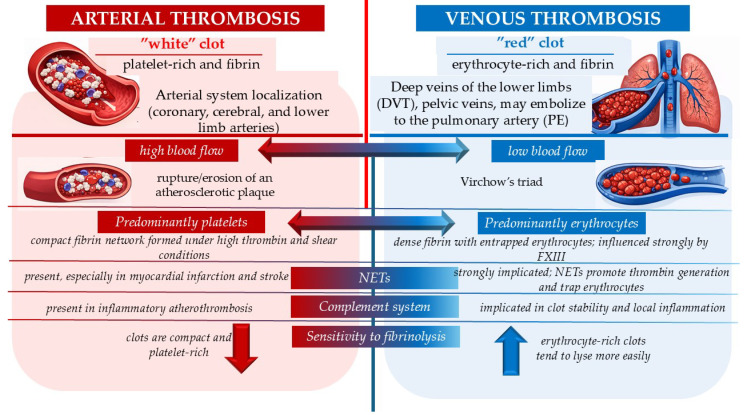
Principal characteristics of arterial and venous thrombosis according to Lippi and Favaloro (2018) [20].

The composition of the two types of thrombi (arterial and venous) has traditionally been considered different because the conditions under which thrombi form and develop, as well as the pathophysiology of thrombus formation in the arterial versus the venous system, do not fully overlap. Thrombi in the arterial system, known as “white” thrombi, are predominantly composed of platelet aggregates, whereas those in the venous system, known as “red” thrombi, are predominantly composed of fibrin and erythrocytes. This dichotomy is only partially true, as studies have shown variations in thrombus composition even within the same vascular system. These variations depend on the time of formation, the underlying cause, pathogenesis, and clot age, as well as several genetic and acquired factors. Nevertheless, distinguishing between the two types of thrombi enables tailored therapeutic management—antiplatelets or anticoagulants [20]. Figure 2 summarizes the characteristics of arterial and venous thrombosis according to Lippi and Favaloro (2018) [20].

Initially, the proteomic analysis method for characterizing the composition of fibrin clots generated from the plasma of patients with VTE was optimized using multiple enzyme digestion filter-aided sample preparation (MED-FASP), applying three different enzymes (LysC, trypsin, and chymotrypsin), together with strong anion exchange (SAX) fractionation combined with LC–MS/MS analysis. The fibrin clot was generated from peripheral blood collected from 4 patients with a median age of 36 years who had experienced VTE (2 patients with isolated DVT and 2 with isolated PE) diagnosed on average 12 months earlier. A total of 476 proteins were identified. The proteins with the highest concentrations were involved in biological processes such as platelet activation (including von Willebrand factor), the coagulation cascade (coagulation factors IX, V, VIII, XII, XIII A chain, XIII B chain, fibrinogen alpha, beta and gamma chains, and prothrombin), regulation of blood coagulation (alpha-2-antiplasmin, antithrombin III, beta-2-glycoprotein 1), fibrinolysis (plasminogen), immune system processes (alpha-2-macroglobulin), and regulation of the protein activation cascade (fibronectin, vitronectin). Moreover, this method was also capable of identifying extracellular vesicles [21].

Starting from the premise that antiphospholipid syndrome is associated with a prothrombotic clot phenotype characterized by a dense fibrin network, a comparative analysis of the plasma composition of fibrin clots was performed between patients with thrombotic antiphospholipid syndrome and those with VTE (with a median of 20 months from diagnosis). The results demonstrated differences in the modulation of complement components, antithrombotic proteins, and platelet proteins [22].

Expanding on their earlier findings, Bryk et al. (2020) examined plasma fibrin clots using proteomic approaches and assessed their association with clot properties in patients with acute PE [23]. A total of 198 proteins were statistically different between patients with acute PE and healthy controls. Increased levels of clot-bound fibrinogen (+14%), apolipoprotein B-100 (+152%), platelet glycoprotein Ib (+884%), and lipopolysaccharide-binding protein (+144%) were identified in the clots of patients with acute PE. Clot-bound histones H3 and H4 were identified exclusively in plasma fibrin clots from acute PE patients. At the same time, plasma fibrin clots from acute PE patients showed lower levels of clot-bound fibronectin (−27%), alpha-2-antiplasmin (−63%), alpha-2-macroglobulin (−67%), FXIII (−76%), histidine-rich glycoprotein (−84%), antithrombin (−73%), von Willebrand factor (−26%), plasminogen (−54%), and prothrombin (−88%) [23].

Clot properties were also assessed in the same study. The median clot lysis time was 112 min, and the median permeation coefficient was 3.83 × 10^−9^ cm^2^. Increased amounts of C-reactive protein (CRP), kininogen-1, protein S, beta-2-microglobulin, and thromboxane-A synthase were associated with lower Ks values, indicating reduced clot permeability [23]. Regarding the variability of fibrin clot composition, an increase in clot-bound FXIII and clot-bound alpha-2-antiplasmin was observed at 3-month follow-up, by 55.1% (from 2.97 mg/g protein to 4.66 mg/g protein) and 16.8% (from 9.4 mg/g protein to 11 mg/g protein), respectively. Moreover, plasma levels of FXIII activity and alpha-2-antiplasmin activity increased from 20.1% at admission to 25.8% at 3-month follow-up, and from 9.1% to 12%, respectively. Fibrin clot lysis time decreased by 17.1%, and fibrin clot permeation increased by 38.5% at 3-month follow-up compared with admission [24]. Table 2 provides a comparative overview of the studies on clot composition.

Platelets are essential components for clot formation. Activated platelets can release hundreds of proteins involved in the inflammation process and thrombosis. Evaluation of plasma signatures of platelet-related protein release in acute PE identified 135 of proteins originated from granule secretion, extracellular vesicles, and membranes. The subtypes, isolated PE or isolated DVT, was studied along with the common form of VTE. A profile consists of 33 proteins that differentiate the acute isolated PE subtype with an AUC of 0.94. Twenty-two of these 33 proteins were found only in acute isolated PE. There are secretory proteins such as urokinase-type plasminogen activator, cathepsin D, C-X-C motif chemokine 1, C-C motif chemokine 3, the antimicrobial protein neutrophil defensin 1, interleukin1α, the extracellular matrix glycoprotein tenascin-X, and platelet-derived growth factor subunit β. There are proteins from shedding or extracellular vesicles, such as endoglin, transferrin receptor protein 1, urokinase plasminogen activator surface receptor, sortilin, and angiotensin-converting enzyme 2. There are also proteins from protein kinases and phosphatases: proto-oncogene tyrosine-protein kinase; mitochondrial proteins: PRDX3, thioredoxin-dependent peroxide reductase; 2,4-dienoyl-CoA reductase, mitochondrial; signaling and adapter proteins: diacylglycerol kinase zeta; MGMT, methylated-DNA-protein-cysteine methyltransferase; DAPP1, dual adapter for phosphotyrosine and 3-phosphotyrosine and 3-phosphoinositide; other metabolism: superoxide dismutase Cu-Zn; phospholipid transfer protein; transcription and translation: eukaryotic translation initiation factor 5A-1 [25].

FXI played a role in the coagulation cascade, but some studies show an association with cardiovascular risk. The role in VTE was evaluated in a proteomic study, part of GMP VTE project, which included 549 patients with acute VTE with a mean age of 59.8 ± 16.5 years and 184 patients at 12 months after VTE. In the acute phase, plasma levels of FXI:C were significantly higher (120.8 ± 33.79%; *p* < 0.001) than at 12 months follow-up (87.2 ± 34.3%). The proteomic analysis showed that 21 proteins were associated with FXI:C in the acute phase. These proteins played a role in cell differentiation and cell adhesion, the immune system, and inflammation. Sixty-six proteins with FXI:C at 12-month follow-up and 7 proteins associated with FXI:C in both moments. The shared proteins played a role in the innate system [26].

### 2.3. Special Clinical Contexts: COVID-19 and Cancer

Coronavirus disease 2019 (COVID-19) is associated with a marked increase in VTE risk. Proteomic studies have identified P-selectin and angiopoietin-like protein 4 (ANGPL4) as independent markers of thrombotic risk and mortality in hospitalized patients.

Fenyves et al. (2021) assessed the relationship between coagulation proteins and VTE in a cohort of 306 COVID-19 patients [27]. Thirty-one biomarker proteins involved in coagulation were selected from available databases (Olink and Somalogic). Among these, COVID-19 patients who developed VTE had increased levels of factor IX, X, p-selectin, plasma, PCR, and vWF and decreased levels of ADAM, antithrombin 13, factor VII, protein C, and prolylcarboxypeptidase on day 0. P-selectin was independently associated with VTE. It was also associated with VTE severity on days 3 and 7, but not on day 0, suggesting its role in delayed endothelial injury. When assessing the predictive capacity of VTE and disease severity in COVID-19-positive patients, the combination of D-dimer and p-selectin values was superior to D-dimers alone (AUC 0.834 vs. AUC 0.783) [27]. The role of p-selectin in the prothrombotic effect of SARS-CoV-2 infection was also investigated in the CRITICAL trial, which evaluated the effect of crisanlizumab (a monoclonal antibody against P-selectin) in COVID-19 patients (NCT04435184) [28].

ANGPTL4 is another protein whose increased plasma concentration has been independently associated with the risk of VTE (95% CI, 1.11–1.98; *p* = 0.008) and mortality (95%CI, 1.17–2.00 per doubling of ANGPTL4; *p* = 0.002) in COVID-19-positive patients hospitalized in intensive care. Its secretion depends on hypoxic conditions in critically ill patients [29].

The post-COVID-19 period is associated with a prothrombotic state. This can be explained by the low plasma level of LRRC15, a receptor for SARS-CoV-2, which has been associated with increased severity in patients with COVID-19 [30].

Lopuhaä et al. (2024) performed proteomic analysis of lung tissue and thrombi in patients with SARS-CoV-2 and VTE (8 patients) compared to influenza (11 patients) using liquid chromatography coupled with mass spectrometry (LC/MS) [31]. Analysis of lung parenchyma samples revealed 356 significantly different proteins, of which 27 were upregulated. Thirty-seven genes were upregulated in patients with COVID-19, of which the urea cycle and aldehyde dehydrogenases pathways were most significant. Thrombus analysis identified 127 different proteins between thrombi formed in COVID infection compared to influenza, 4 upregulated and 87 downregulated, involving inflammatory response and platelet activity. Fifty-four proteins were different in the structure of thrombi formed in situ versus those embolized. Endothelial analysis found 149 modified proteins and 2 pathways involving DHCR24 and CSDE1 signaling [31].

In oncology, proteomic profiling has revealed VTE-associated signatures in non–small cell lung cancer (NSCLC), including serum amyloid A1, S100A8, and Hp, suggesting potential utility for early risk stratification. Proteomic studies have identified 280 proteins that are expressed differently (42 were upregulated, and 238 were downregulated) with DIA-MS in patients with NSCLC who had developed VTE. These proteins are involved in inflammation, acute phase response, cytokine production, or neutrophil migration. Five of these proteins, including serum amyloid A-1, protein S100A8, lipopolysaccharide-binding protein, Hp, and lactate dehydrogenase B showed significant changes and are candidates as biomarkers for the early identification of VTE in NSCLC (with the AUC of 0.8067; 0.8308; 0.7767; and 0.8533, respectively) [32]. Table 3 provides a comparative overview of the studies analyzing specific clinical contexts associated with VTE.

### 2.4. Proteomic Differences Between VTE Phenotypes and Endotypes

Although PE and DVT are traditionally viewed as manifestations of a single disease spectrum, accumulating evidence supports the existence of distinct biological phenotypes. The well-known examples are increased body-mass index (BMI), estrogen secretion, and the factor V Leiden paradox, which are associated with a significantly higher risk for DVT than for PE.

The Genotyping and Molecular Phenotyping Venous Thromboembolism (GMP-VTE) project, a multicenter cohort study, was designed to analyze genes and targeted proteins involved in VTE during the acute phase and follow-up, providing insights into the pathophysiology of the disease. Six hundred sixty-three patients with a mean age of 60.3 ± 15.9 years, diagnosed with acute VTE were evaluated. One hundred seventeen of them had only isolated PE, and 188 only isolated DVT. Proteomic analysis identified proteins involved in modulating immune and inflammatory response, cell adhesion, and coagulation [33]. The researchers analyzed the existence of different phenotypes between isolated PE, VTE, and isolated DVT after excluding patients with neoplasms. The results were then validated in a cohort of 5778 patients with a 2.9-year follow-up. The group of patients with isolated PE was characterized by a lower BMI, absence of thrombophilia, fewer surgical interventions, a low proportion of carriers of factor V Leiden or prothrombin G20210A gene mutations, but a higher prevalence of atherosclerotic diseases, heart failure, and chronic pulmonary diseases. Smoking was twice as frequently repeated in the group with isolated DVT. Proteins involved in modulating immuno-inflammation (CD244, AZU1, SRC, SAA4, IL-4, IL-10), oxidative stress response (antioxidant HO-1), or pulmonary surfactant loss (lung-specific surfactant protein SCGB3A2) were shared by PE phenotypes. The specific proteomic profile of isolated PE included five proteins: IFN gamma, glial cell line-derived neurotrophic growth factor, polypeptide N-acetylgalactosaminyltransfersase 3, peptidyl arginine deiminase type-2, and IL-15 receptor subunit alpha [34]. In conclusion, the results showed that isolated PE is characterized by a specific proteomic signature enriched in proteins related to immune–inflammatory modulation, oxidative stress, and pulmonary injury.

Starting from the obesity paradox also observed in VTE, where obesity increases the risk of disease development but appears to be protective against recurrence and mortality, Ten Cate et al.(2021). analyzed circulating proteins in samples collected from 657 patients with VTE enrolled in the GMP-VTE project [35]. Obese patients showed a higher prevalence of VTE but a lower incidence of VTE recurrence and death (incidence rate ratio = 0.41; 95% CI: 0.23–0.74; *p* = 0.003). The body mass–associated protein signature in patients with VTE comprised 11 proteins (CLEC4C, FABP4, FLT3LG, IL-17C, LEP, LYVE1, MASP1, ST2, THBS2, THBS4, and TSLP). This protein signature was not associated with the obesity paradox. However, leptin showed a negative correlation with VTE recurrence or death, corresponding to a 34% risk reduction per one standard deviation increase in leptin concentration [35].

The heterogeneity of patients with VTE in the GMP-VTE project was also analyzed. Four endotypes were identified based on 58 variables, with the most influential being clinical characteristics, acute-phase protein signatures, recurrence of thromboembolic events, and mortality. Endotype 1 (n = 300) comprised elderly patients with multiple comorbidities (increased BMI, chronic kidney and liver disease, arterial hypertension, congestive heart failure), characterized by elevated levels of cytokines and inflammatory proteins, and having the poorest prognosis. Endotype 4 (*n* = 127) included predominantly men with DVT, provoking risk factors, and VTE history, showing increased metalloproteinase activity and elevated levels of proteins involved in cell adhesion. Endotype 3 (*n* = 57) consisted mainly of young women with DVT and provoking risk factors, and was characterized by elevated levels of proteins involved in coagulation and angiogenesis. Both endotypes 3 and 4 had a moderate prognosis for recurrence of thromboembolic events and death compared with endotype 2, which showed the most favorable prognosis [36].

Sex-specific biomarkers have also been proposed, such as lipopolysaccharide-binding protein (LBP), which was associated with increased DVT risk in women. Jensen et al. (2022) conducted an untargeted proteomic analysis consisting of a discovery phase based on data collected in the Tromsø Study, a prospective case-control study, and a validating phase [37]. The aim was to identify candidate biomarkers for differentiating between isolated PE and isolated DVT, stratified by sex. The discovery study included 100 patients with VTE with a median age of 69.2 years and 52% women. Fifty-five cases presented isolated DVT, and 25 cases presented isolated PE. It was found that elevated LPB was a candidate biomarker for developing DVT in women over a period of 3 years from blood sampling. In the validation study conducted on 410 patients with VTE, the association between elevated LPB and DVT in women was smaller, but significant after adjustment for BMI and PCR levels. The increase of 1 SD in plasma LPB levels doubles the OR for DVT in women when the follow-up was 3 years [37]. One of the limitations of this study was the unknown influence of oral contraceptive use. To overcome this limitation, Granholm et al. (2022) include only men with PE in a non-targeted proteomic analysis using LC-MS/MS (eight men with PE vs. eight controls) [38]. One hundred thirty-seven proteins were identified in all samples, and 13 proteins were significantly different. Proteins with elevated levels of PE were complement component 9, complement factor H, and leucine-rich alpha2-glycoprotein, and those with low levels were apolipoprotein C-III, carboxylic ester hydrolase, antithrombin-III, procollagen C-endopeptidase enhancer, serpinpeptidase inhibitor clade A, member 4, carboxypeptidase B2, afamin, serpinpeptidase inhibitor clade A member 5, coagulation factor XII, and N-acetylmuramoyl-L-alanine amidase. Those proteins are known to modulate inflammation, atherosclerosis, and thrombosis processes [38]. Table 4 provides a comparative overview of the studies analyzing the proteomic signatures associated with isolated PE or isolated DVT.

### 2.5. Another Source for Proteomic Analysis

Exhaled breath condensate (EBC) is a new method for identifying biomarkers among small-molecule compounds. EBC can be obtained quickly, non-invasively, and easily from most patients, including children and intubated, mechanically ventilated patients. Samples are collected by cooling exhaled air and capturing the resulting condensate. Improvements in collection devices (RTube, TurboDECCS, EcoScreen), the use of LC/MS, and the optimization of sample-processing protocols have provided valuable insights into the proteomic analysis of EBC for identifying diagnostic and prognostic biomarkers in lung diseases, particularly lung cancer [39].

In 2021, Gade et al. conducted a pilot study to analyze the impact of EBC sample-collection temperature on sample quality and proteomic analysis [40]. EBC was collected from seven Danish Landrace pigs before and 2.5 h after the induction of intermediate-risk acute PE, at two different temperatures: −80 °C and −21 °C. The mean concentration of proteins present in the EBC was 5.85 ± 0.93 µg/mL, with no differences between the two collection temperatures. Analysis by nano-liquid chromatography coupled with mass spectrometry (nLC-MS/MS) identified 254 proteins across all cellular compartments. Of these, 131 proteins were present in all EBC samples. The levels of five proteins changed significantly before and after PE induction. Two proteins (P81245 and P30086) were approximately 29-fold higher after PE induction, while the levels of the other three proteins (P06753-4, P31949, P13797-3) decreased after PE. The collection temperature of −80 °C was shown to be optimal for the proteomic analysis of EBC in pigs [40].

The same research team continued the proteomic analysis using nLC-MS/MS to identify potential PE biomarkers in the EBC of 18 Danish Landrace pigs (14 with intermediate–high-risk acute PE and 4 control pigs without PE). Six samples were collected from each pig—before PE induction, 30 min after, and 2.5 h after the induction of acute PE. The analysis was performed in two phases. The first phase was the discovery phase of potential biomarkers, in which differences in EBC protein expression were identified using Student’s *t*-test. In the second phase, prediction and differential expression analysis models were applied, and corrections for multiple comparisons were made to qualify biomarkers for PE [41].

The mean protein concentration in EBC was 2.83 ± 0.08 µg/mL in the PE group and 3.17 ± 0.20 µg/mL in the control group, with no statistically significant difference between the two groups. A total of 897 proteins were identified in the EBC samples. In the discovery phase, the levels of 145 proteins changed after PE induction. Fifty proteins, mostly from the cytosol or cytoplasm, were increased (fold change 1.3 to 17.1) after PE induction and showed biological connections among them. Albumin was one of the most increased proteins (14-fold). Ninety-five proteins, mostly cytosolic or cytoplasmic (ninety recognized by STRING), showed decreased levels after PE induction, with fold changes ranging from 0.1 to 0.8; the lowest was NGAL (neutrophil gelatinase–associated lipocalin) (↓0.3×) [41].

Multivariate analysis identified 30 proteins modified after PE induction and based on these results, the final prediction models were created: (i) increased expression of ACTG1 and TUBA1B decreases the probability of PE, while increased expression of DSG1 increases the probability of PE (by comparing EBC samples collected 30 min after PE induction vs. control, 5.5% misclassification errors, ROC AUC 0.93, cutoff value of 0.53); (ii) increased expression of RPLP2, LCN2, and IL36G decreases the likelihood of EP, while increased expression of ALB and IGHA1 increases the likelihood of PE (by comparing EBC samples collected 2.5 h after PE induction vs. before induction, 18.5% misclassification errors, ROC AUC 0.77, cutoff value of 0.45 with a sensitivity of 0.85 and a specificity of 0.85); and (iii) increased expression of A2ML1, HSP90AA1, GGCT, SERPINB7, PGK1, C3, HBA, HSPB1, ARG1, S100A8, RPLP2, CTSD, RPSA, GSTP1, TXN, CFL1, EEF2, DSP, H1-5, TYMP, TGM1, CALML3, SERPINB3, MYH9, LGALS7, SERPINB4, VCP, EIF6, EPPK1, TPI1, CALM1, EEF1A1P5, TUBA1B, LCN2, FABP5, PRDX1, PKP1, H2AC20, PARK7, IL36G, and CALML5 decreases the probability of PE, while increased expression of LDHA, CSTA, IGHA1, TPM3, ALB, TF, UBA52, PIP, AZGP1, LCN1, HAL, BLMH, CDSN, NCCRP1, SERPINB12, and CPA4 increases the probability of EP (by comparing EBC samples collected 30 min after PE induction vs. before induction, 3.5% misclassification errors, ROC AUC 0.99, cutoff value of 0.51) [41].

The first proteomic analysis study of EBC for identifying diagnostic biomarkers of acute PE in humans included 77 participants: 28 patients with confirmed PE and 49 patients without PE. A total of 928 proteins were identified. Among these, HSPA5 (a cellular stress marker), PEBP1, and SFTPA2 (a surfactant protein associated with alveolar injury) were significantly increased, while POF1B, EPPK1, PSMA4, ALDOA, and CFL1 were significantly decreased in patients with acute PE. EBC may be a valuable source of biomarkers for diagnosing PE; however, the extremely low protein concentrations require further optimization of analytical methods [42]. Table 5 provides a comparative analysis between proteomic studies in EBC.

In conclusion, EBC represents a novel, non-invasive source of protein biomarkers, providing access to molecular signatures that could enhance early diagnosis, risk stratification, and disease monitoring. Experimental and clinical studies have demonstrated the feasibility of proteomic analysis of EBC in PE, identifying proteins related to cellular stress and alveolar injury. However, extremely low protein concentrations remain a major technical limitation that requires further methodological development and optimization.

Another accessible source for proteomic analysis is urine. Increased proteolytic activity associated with VTE leads to the release of small protein fragments that can pass through the renal filtration barrier and reach the urine. Von Zur Mühlen et al. (2016) identified 62 peptides associated with DVT in urine samples from patients using capillary electrophoresis coupled with mass spectrometry (CE-MS) [43]. Elevated levels of fragments derived from type III collagen, apolipoprotein A1, uromodulin, osteopontin, fibrinogen, gelsolin, ion protease homolog, peptidoglycan recognition protein 1, plasma protease C1 inhibitor, POTE ankyrin domain family member F, receptor-type tyrosine-protein phosphatase U, retinol-binding protein 4, and WD repeat–containing protein 59 were observed. The peptide-based model demonstrated an AUC of 0.90, with 100% specificity and 83% sensitivity for diagnosing DVT [43].

### 2.6. Prospective Future Incidental VTE

Prospective studies indicate that proteomic changes may precede the clinical onset of VTE. Within the Tromsø Study, Jensen et al. (2018) identified a panel of 46 proteins altered before thromboembolic events occurred using TMT-SPS-MS3. Transthyretin, vitamin K–dependent protein Z, and protein/nucleic acid deglycase DJ-1 demonstrated the strongest predictive value for future incidental VTE, with the values of *p* of 0.00015, 0.0018, and 0.0055, respectively [44]. The study showed that these proteins also interact with each other to increase the risk [44].

Another study evaluated biomarkers for the prognosis of PE. The study was divided into a discovery phase that included DIA-MS and antibody analysis and a verification phase using ELISA. A total of 207 proteins were identified in patients with PE, and 70 of these were correlated with high risk. The role of the proteins was in modulating coagulation, acute inflammatory response, and receptor-mediated endocytosis. Three proteins were validated by ELISA, SAA (serum amyloid A-1), S100A8 (calprotectin), and TNC (tenascin-C), as biomarkers for high-risk PEwith AUC of 0.882, 0.788, and 0.795, respectively [45].

### 2.7. Recent Perspectives

Recent proteome-wide association studies have identified novel proteins associated with VTE, including markers involved in atherosclerosis, shear stress, and endothelial function. Although some studies have failed to identify robust plasma proteomic signatures, the cumulative evidence supports the substantial potential of proteomics in advancing precision medicine for VTE.

Kong et al. (2025) conducted a linear and non-linear proteome-wide association study to advance understanding of VTE [46]. Initially, 237 Olink proteins and 408 Soma Scan proteins were selected from 378,474 patients (22,102 with VTE and 356,371 controls) from the UK Biobank. 43 proteins associated non-linearly with VTE were identified, of which 26 had not been previously reported, including TEK, CCL25, and VSIG2. The results were subsequently tested in a prospective cohort in the UK Biobank Pharma Proteomics Project. Eight proteins were associated non-linearly with VTE, including ULBP2, IL18BP, MAN1A2, CCL25, ICAM2, LGALS4, VSIG2, and ABO. The proteins were involved in the atherosclerotic process, shear stress, and endothelial properties [46].

Smit et al. (2025) attempted to find a plasma proteomic signature associated with the occurrence of thromboembolic events using MS [47]. They included patients with atrial fibrillation and pulmonary thromboembolism treated with anticoagulant therapy with antivitamin K, patients with a history of VTE, patients with acute cerebral venous sinus thrombosis, and patients with SARS-CoV-2 infection. They analyzed 434 plasma proteins and compared them with laboratory parameters, but no association was identified [47].

### 2.8. Limitations

Although recent studies have reported promising findings regarding the application of proteomic profiling in VTE, several important limitations must be acknowledged. First, the relative novelty of this research area has hindered the establishment of a standardized and universally accepted proteomic analysis protocol. Consequently, substantial methodological heterogeneity exists among studies, including differences in the timing of biological sample collection (prior to or following the initiation of anticoagulant therapy), procedures for sample storage and processing, and variability in inclusion and exclusion criteria and the selection of appropriate control groups.

Second, the available evidence is derived from studies that have used diverse biological specimens and employed various analytical platforms and techniques to quantify protein expression levels. This lack of methodological uniformity limits the comparability of results across studies and may contribute to inconsistencies in reported findings.

Third, most published investigations have been conducted in relatively small study populations, which may reduce statistical power and limit the generalizability of the results. Taken together, these limitations warrant careful interpretation of the existing data and highlight the need for future large-scale, well-designed studies that incorporate standardized methodologies and adequately address these sources of variability.

## 3. Conclusions

Despite the availability of diagnostic and management algorithms, acute VTE remains associated with a significant global burden. In recent years, proteomic research has contributed substantially to a better understanding of VTE by shedding light on the molecular mechanisms involved in its development, clinical expression, and outcomes. The findings reviewed here suggest that VTE cannot be explained solely by a linear activation of the coagulation cascade. Instead, it appears to result from a complex interaction between inflammation, immune responses, endothelial dysfunction, platelet activity, and changes in fibrin clot composition.

Several studies have consistently reported alterations in acute-phase proteins, transport proteins, complement components, and regulators of fibrinolysis. These observations support the central role of thromboinflammation in VTE pathophysiology. Proteins such as Hp, Fn, pentraxin-3, complement factor H–related proteins, and platelet-derived mediators have been repeatedly associated with disease presence, severity, and the risk of recurrence. Importantly, changes detected in the circulation often reflect molecular alterations within the thrombus itself, which strengthens their biological relevance.

**Figure 3:**
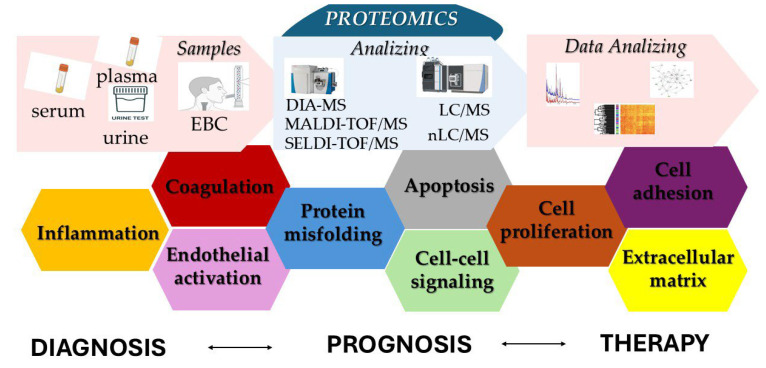
Proteomic analysis in VTE.

One of the key contributions of proteomic research is the emerging distinction between PE and DVT as partially separate biological entities. Data from large cohort studies indicate that isolated PE is characterized by a specific molecular profile, with enrichment of immune-inflammatory, oxidative stress–related, and pulmonary-associated proteins. This challenges the traditional view of VTE as a single, uniform disease spectrum. In addition, the identification of sex-specific and phenotype-specific biomarkers highlights the heterogeneity of VTE and supports the need for more individualized risk assessment strategies. Proteomic studies performed in specific clinical settings, such as COVID-19 and cancer, have further revealed distinct thrombotic patterns. These signatures appear to reflect disease-specific mechanisms, including endothelial injury, hypoxia-related pathways, and persistent immune activation. In such complex contexts, proteomics may offer added value beyond conventional clinical and laboratory markers (Figure 3 and Table S2).

Overall, the available evidence supports proteomics as a promising tool for advancing precision medicine in VTE. At present, differences in study design, relatively small sample sizes, and the limited availability of external validation restrict direct clinical application. However, integrating proteomic data with clinical, genetic, and imaging information may substantially improve disease characterization. Future prospective and standardized multi-omics studies will be essential to validate candidate biomarkers and to support more personalized diagnostic and therapeutic approaches in Venous Thromboembolism.

## Figures and Tables

**Table 1:** Comparative overview of studies analyzing inflammation in VTE.

	Objective	Populations	Design	Methods	Biological Sample	Number of Proteins Identified	Key Proteins	Main Findings
Insenser et al., 2014 [14]	Identification of circulating proteomic alterations associated with a prothrombotic phenotype	Humans	Exploratory proteomic study	2-DE + MALDI-TOF-MS	Serum/plasma	Multiple differentially expressed proteins (not a fixed panel)	Acute-phase proteins, transport proteins, and inflammatory markers (e.g., Hp, apolipoproteins)	Altered serum proteomic profile reflects interaction between inflammation and thrombosis
Zhang et al., 2018 [15]	Identification of diagnostic serum biomarkers for acute PE	Humans	Two-stage study: discovery + validation	2-DE + MALDI-TOF-MS; ELISA validation; immunohistochemistry	Serum and pulmonary tissue	8 candidate proteins identified in the discovery phase	Hp, ITIH4, clusterin, LRG1, RBP4, A1M/Bikunin	Hp significantly increased in acute PE and was expressed within pulmonary thrombi

**Table 2:** Comparative overview of the three studies on the clot composition.

	Population	Methods	Identified Proteins	Increased Proteins in the Clot	Decreased Proteins in the Clot	Fibrin Clot Properties
Stachowicz et al., 2017 [21]	4 patients with chronic VTE (>12 months)	MED-FASP + LC-MS/MS	476	vWF, FXII, FXIII, fibrinogen chains, prothrombin	Few variations	
Bryk et al., 2020 [23]	Acute PE vs. control	LC-MS/MS + functional evaluation Ks, clot lysis time	198 statistics differentiate proteins	fibrinogen (+14%), ApoB-100 (+152%), GPIb (+884%), LBP (+144%), histone H3/H4	fibronectin (−27%), α2-antiplasmin (−63%), α2-macroglobulin (−67%), FXIII (−76%), HRG (−84%), antithrombin (−73%), plasminogen (−54%)	Clot lysis time = 112 min; Ks = 3.83 × 10^−9^ cm^2^
Zabczyk et al., 2021 [24]	Patients with VTE (PE or DVT) acute and 3-month follow-up	No proteomic analysisFXIII, α2-antiplasmin and clot properties	-	Clot-bound FXIII ↑ (+55% la 3 luni); α2-antiplasmin ↑	FXIII and α2-antiplasmin decreased in the acute phase, and normal values at follow-up	At 3 months: lysis time ↓ 17%, permeabily ↑ 38.5%

**Table 3:** A comparative overview of the four proteomic studies analyzing specific clinical contexts associated with VTE.

	Objective	Populations	Design	Methods	Biological Sample	Number of Proteins Identified	Key Proteins	Main Findings
Fenyves et al., 2021 [27]	To identify early plasma biomarkers associated with VTE in COVID-19	306 COVID-19–positive patients presenting with respiratory distress	Prospective observational cohort with targeted proteomic analysis	High-throughput proteomics using Olink Explore 1536 and SomaScan platforms	Plasma samples collected at admission and during hospitalization	**1**	P-selectin identified as an early and independent marker of VTE; improves prediction when combined with D-dimer	Early detection of thromboembolic complications in COVID-19. P-selectin rises early in VTE and enhances diagnostic performance beyond D-dimer alone
Gisby et al., 2022 [30]	To identify molecular signatures of COVID-19 severity and persistent pro-thrombotic signals using multi-omics	70 end-stage kidney disease (ESKD) patients on hemodialysis with COVID-19, plus controls	Longitudinal multi-omics study with pre-infection, acute, and convalescent sampling	SomaScan plasma proteomics (6323 proteins) combined with PBMC RNA sequencing	Plasma, PBMCs (RNA-seq), and flow cytometry samples collected longitudinally	1	Decreasing plasma LRRC15 associated with severe disease; persistent post-COVID upregulation of coagulation and platelet pathways (e.g., PF4)	Severity stratification and long-term thrombotic risk after COVID-19. COVID-19 induces prolonged pro-thrombotic molecular changes lasting months after recovery
Lopuhaä et al., 2024 [31]	To characterize proteomic differences underlying increased venous thromboembolism in SARS-CoV-2–infected lung tissue	8 COVID-19 and 11 influenza autopsy cases with pulmonary thrombi	Postmortem comparative tissue proteomics study	LC–MS with laser capture microdissection	Formalin-fixed paraffin-embedded lung tissue, isolated endothelium, and isolated thrombi	-	Upregulation of liver metabolism pathways (e.g., arginase); reduced platelet activation pathways in COVID-19 thrombi	Differentiation between venous thromboembolism and in situ pulmonary thrombosis. Proteomic evidence supports increased venous thromboembolism in COVID-19 lungs
Liu et al., 2023 [32]	To discover plasma protein biomarkers for VTE in NSCLC patients	35 NSCLC patients (20 with VTE, 15 without VTE)	Case–control proteomics study	DIA-MS	Citrated plasma collected within 3 months of VTE diagnosis	5	Five candidate biomarkers identified: SAA1, S100A8, LBP, HP, LDHB (AUC up to 0.85)	Early diagnosis of cancer-associated VTE. Specific inflammatory and acute-phase proteins discriminate VTE in NSCLC patients

**Table 4:** A comparative overview of the studies analyzing the proteomic signatures associated with isolated PE or isolated DVT.

	Objective	Populations	Design	Methods	Biological Sample	Number of Proteins Identified	Key Proteins	Main Findings
Ten Cate et al., 2021 [34]	To determine whether isolated PE has a distinct acute-phase plasma proteomic signature compared to DVT-associated PE and isolated DVT	532 patients with imaging-confirmed acute VTE (96 isolated PE, 276 DVT-associated PE, 160 isolated DVT)	Multicenter prospective cohort with cross-sectional acute-phase proteomic comparison and external validation	Targeted high-throughput proteomics (Olink proximity extension assay; 444 proteins)	EDTA plasma collected during acute diagnostic workup for VTE	Five isolated PE-specific proteins identified suggesting noncanonical pathways	IFN-γ, GDNF, GALNT3, PADI2, IL-15Rα	Isolated PE shows a distinct proteomic signature linked to inflammatory, pulmonary, and atherosclerotic pathways
Jensen et al., 2022 [37]	To identify and validate predictive biomarkers for future VTE, with distinction between DVT and PE and assessment of time-dependent attenuation	General population cohort (Tromsø Study); 100 cases/100 controls (discovery) and 410 VTE cases/834 controls (validation)	Population-based prospective cohort with discovery and nested case–control validation	Untargeted MS-based proteomics (TMT labeling) for discovery; ELISA for validation	EDTA plasma collected at baseline, years before incident VTE	1	Lipopolysaccharide-binding protein (LBP) identified as a predictive biomarker for DVT in women, with sex- and phenotype-specific effects	Elevated LBP predicts near-term DVT risk in women; effects differ by sex and VTE subtype
Granholm et al., 2022 [38]	To evaluate the feasibility of label-free quantitative proteomics for identifying potential biomarkers in acute PE	16 male patients: 8 with CTPA-confirmed acute PE and 8 symptomatic CTPA-negative controls	Exploratory, case–control feasibility study	Label-free LC–MS/MS after high-abundance protein depletion	Citrated plasma collected in the acute phase (before anticoagulation) and at ≥12-month follow-up	13 proteins significantly altered in acute PE, including complement proteins (e.g., Complement C9, Factor H) and coagulation-related proteins	Complement C9, Factor H	Proteomic profiling can detect acute PE–related protein changes and monitor normalization over time

**Table 5:** Proteomic analysis from exhaled breath condensate.

	Objective	Populations	Design	Methods	Sampling	Protein Concentrations	Number of Proteins Identified	Key Proteins	Main Findings
Gade et al., 2021 [40]	Testing collection temperatures and evaluating the feasibility of proteomics	7 female Danish Landrace pigs with induced intermediate–high risk acute PE	Experimental, porcine model	LFQ nLC–MS/MS	Time point: Before and ~2 h after PE Collection device: RTube Vent at −21 °C vs. −80 °C Temp: −21 °C vs. −80 °C EBC volume: −80 °C: 1.78 mL vs. −21 °C: 0.71 mL	~5.85 µg/mL	254	Minimal differences (mainly volume, not composition)	−80 °C provides a higher volume without altering the proteome
Gade et al., 2021 [41]	Identification of PE biomarkers in EBC	14 female Danish Landrace pigs with induced intermediate–high risk acute PE vs. 4 control Danish Landrace pigs	Experimental, porcine model	LFQ nLC–MS/MS	Time point: Before, 30 min, and 2.5 h after PE Collecting device: RTube Vent connected to a ventilator Temp: −80 °C (standard) EBC volume: 1–3 mL	~6 µg/mL	897	145 proteins altered post-PE Albumin ↑14×; 49 proteins ↑ NGAL ↓0.3×; 95 proteins ↓	EBC contains potential biomarkers for PE
Gade et al., 2023 [42]	Identification of PE biomarkers in human EBC	28 patients with PE + 49 controls	Clinical, human	LFQ nLC–MS/MS	Single session during clinical evaluation Collecting device: RTube™ for spontaneous breathing Temp: −80 °C pre-cooled EBC volume: 3–7 mL → then evaporated	<0.5 µg/mL (very low)	928	8 candidate proteins (3 ↑, 5 ↓): HSPA5, PEBP1, SFTPA2 ↑ POF1B, EPPK1, PSMA4, ALDOA, CFL1 ↓	Human EBC may contain PE biomarkers

## Data Availability

No new data were created or analyzed in this study.

## Supplementary Materials

The following supporting information can be downloaded at: https://www.mdpi.com/article/10.3390/medsci14010094/s1, Table S1: Studies included in the analysis; Table S2: Relevant proteins identified as biomarkers in venous thromboembolism. References [6,7,8,14,15,16,17,18,19,21,22,23,24,25,26,27,29,30,31,32,33,34,35,36,37,38,40,41,42,43,44,45,46,47] are cited in the Supplementary Materials.

## Abbreviations

The following abbreviations are used in this manuscript:

2-DE 2DGE	Two-Dimensional Gel Electrophoresis
ANGPTL4	Angiopoietin-Like Protein 4
AUC	Area Under the Curve
BMI	Body Mass Index
CFHR5	Complement Factor H–Related Protein 5
CI	Confidence Interval
COVID-19	Coronavirus Disease 2019
CPR	C-Reactive Protein
CTPA	Computed Tomography Pulmonary Angiography
DBP	Vitamin D–Binding Protein
DIA-MS	Data-Independent Acquisition Mass Spectrometry
DOACs	Direct Oral Anticoagulants
DVT	Deep Vein Thrombosis
EBC	Exhaled Breath Condensate
ELISA	Enzyme-Linked Immunosorbent Assay
Fn	Fibronectin
FXI	Coagulation Factor XI
FXIII	Coagulation Factor XIII
GMP-VTE	Genotyping and Molecular Phenotyping of Venous Thromboembolism
HO-1/HMOX1	Heme Oxygenase 1
LC-MS/MS	Liquid Chromatography–Tandem Mass Spectrometry
LFQ	Label-Free Quantification
LRRC15	Leucine-Rich Repeat Containing Protein 15
MALDI-TOF MS	Matrix-Assisted Laser Desorption/Ionization Time-of-Flight Mass Spectrometry
MED-FASP	Multiple Enzyme Digestion–Filter-Aided Sample Preparation
NETs	Neutrophil Extracellular Traps
NGAL/LCN2	Neutrophil Gelatinase–Associated Lipocalin
nLC-MS/MS	Nano–Liquid Chromatography–Tandem Mass Spectrometry
NSCLS	Non–Small Cell Lung Cancer
OR	Odds Ratio
PBMC	Peripheral Blood Mononuclear Cells
PE	Pulmonary Embolism
PESI	Pulmonary Embolism Severity Index
RNS-seq	RNA Sequencing
ROC	Receiver Operating Characteristic
SARS-CoV-2	Severe Acute Respiratory Syndrome Coronavirus 2
SAX	Strong Anion Exchange
SELDI-TOF MS	Surface-Enhanced Laser Desorption/Ionization Time-of-Flight Mass Spectrometry
sPESI	Simplified Pulmonary Embolism Severity Index
TF	Tissue Factor
TMT	Tandem Mass Tag
TMT-SPS-MS3	Tandem Mass Tag–Synchronous Precursor Selection–MS3
TTR	Transthyretin
vWF	von Willebrand Factor
VTE	Venous thromboembolism
