# Supplementary material for: Proteomic Insights into Venous Thromboembolism
Source: Med Sci (Basel). 2026 Feb 15;14(1):94. doi: 10.3390/medsci14010094 (PMC12922095; doi:10.3390/medsci14010094)
Supplement: Supplementary file 1 [file medsci-14-00094-s001.zip › medsci-4159159-supplementary.pdf]

Supplementary Materials: Proteomic Insights into Venous Thromboembolism

Oana – Mădălina Manole, Brîndușa Alina Petre and Viviana Onofrei

Table S1. Studies included in the analysis.

Proteomic studies included in qualitative analysis
1. Ganesh, S.K.; Sharma, Y.; Dayhoff, J.; Fales, H.M.; Van Eyk, J.; Kickler, T.S.; Billings, E.M.; Nabel, E.G. Detection of venous thromboembolism by proteomic serum biomarkers. PLoS One 2007,2(6),e544. doi: 10.1371/journal.pone.0000544.
2. Li, S.Q.; Yun, J.; Xue, F.B.; Bai, C.Q.; Yang, S.G.; Que, H.P.; Zhao, X.; Wu, Z.; Wang, Y.; Liu, S.J. Comparative proteome analysis of serum from acute pulmonary embolism rat model for biomarker discovery. J Proteome Res 2007,6(1),150-9. doi:10.1021/pr0603102
3. Insenser, M.; Montes-Nieto, R.; Martínez-García, M.Á.; Durán, E.F.; Santiuste, C.; Gómez, V.; Kline, J.A.; Escobar-Morreale, H.F.; Jiménez, D. Identification of reduced circulating haptoglobin concentration as a biomarker of the severity of pulmonary embolism: a nontargeted proteomic study. PLoS One 2014,9(6),e100902. doi: 10.1371/journal.pone.0100902.
4. Zhang, Y.X.; Li, J.F.; Yang, Y.H.; Huang, K.; Miao, R.; Zhai, Z.G.; Wang, C. Identification of haptoglobin as a potential diagnostic biomarker of acute pulmonary embolism. Blood Coagul Fibrinolysis 2018,29(3),275-281. doi:10.1097/MBC.0000000000000715.
5. Hong, M.; Zhang, X.; Hu, Y.; Wang, H.; He, W.; Mei, H.; Yu, J.; Guo, T.; Song, S. The potential biomarkers for thromboembolism detected by SELDI-TOF-MS. Thromb Res 2009, 123(3), 556-64. doi: 10.1016/j.thromres.2008.05.019
6. Stachowicz, A.; Siudut, J.; Suski, M.; Olszanecki, R.; Korbut, R.; Undas, A.; Wiśniewski, J.R. Optimization of quantitative proteomic analysis of clots generated from plasma of patients with venous thromboembolism. Clin Proteomics 2017, 14:38. doi: 10.1186/s12014-017-9173-x.
7. Stachowicz, A.; Zabczyk, M.; Natorska, J.; Suski, M.; Olszanecki, R.; Korbut, R.; Wiśniewski, J.R.; Undas, A. Differences in plasma fibrin clot composition in patients with thrombotic antiphospholipid syndrome compared with venous thromboembolism. Sci Rep 2018, 8(1), 17301. doi: 10.1038/s41598-018-35034-x.
8. Bryk, A.H.; Natorska, J.; Ząbczyk, M.; Zettl, K.; Wiśniewski, J.R.; Undas, A. Plasma fibrin clot proteomics in patients with acute pulmonary embolism: Association with clot properties. J Proteomics. 2020, 229,103946. doi:10.1016/j.jprot.2020.103946.
9. Baidildinova, G.; Ten Cate, V.; Nagler, M.; Panova-Noeva, M.; Rapp, S.; Köck, T.; Prochaska, J.H.; Heitmeier, S.; Gerdes, C.; Schwers, S.; Konstantinides, S.V.; Münzel, T.; Espinola-Klein, C.; Lackner, K.J.; Spronk, H.M.N.; Ten Cate, H.; van der Meijden, P.E.J.; Leineweber, K.; Wild, P.S.; Jurk, K. Subtype-specific plasma signatures of platelet-related protein releasate in acute pulmonary embolism. Thromb Res 2022, 220, 75-87. doi: 10.1016/j.thromres.2022.10.005

10. Pallares Robles, A.; Ten Cate, V.; Schulz, A.; Prochaska, J.H.; Rapp, S.; Koeck, T.; Panova-Noeva, M.; Heitmeier, S.; Schwers, S.; Leineweber, K.; Seyfarth, H.J.; Opitz, C.F.; Spronk, H.; Espinola-Klein, C.; Lackner, K.J.; Münzel, T.; Andrade-Navarro, M.A.; Konstantinides, S.V.; Ten Cate, H.; Wild, P.S. Association of FXI activity with thrombo-inflammation, extracellular matrix, lipid metabolism and apoptosis in venous thrombosis. Sci Rep 2022, 12(1), 9761. doi:10.1038/s41598-022-13174-5.
11. Fenyves, B.G.; Mehta, A.; Kays, K.R.; MGH COVID-19 Collection & Processing Team; Goldberg, M.B.; Hacohen, N.; Filbin, M.R. Plasma P-selectin is an early marker of thromboembolism in COVID-19. medRxiv [Preprint]. 2021, 2021.07.10.21260293. doi: 10.1101/2021.07.10.21260293. Update in: Am J Hematol 2021, 96(12), E468-E471. doi: 10.1002/ajh.26372.
12. Gisby, J.S., Buang, N.B., Papadaki, A. et al. Multi-omics identify falling LRRC15 as a COVID-19 severity marker and persistent pro-thrombotic signals in convalescence. Nat Commun 13, 7775 (2022).
13. Lopuhaä, B.V.; Guzel, C.; van der Lee, A.; van den Bosch, T.P.P.; van Kemenade, F.J.; Huisman, M.V.; Kruip, M.J.H.A.; Luider, T.M.; von der Thüsen, J.H. Increase in venous thromboembolism in SARS-CoV-2 infected lung tissue: proteome analysis of lung parenchyma, isolated endothelium, and thrombi. Histopathology 2024, 84(6), 967-982. doi: 10.1111/his.15143.
14. Liu, Y.; Gao, L; Fan, Y.; Ma, R.; An, Y.; Chen, G.; Xie, Y. Discovery of protein biomarkers for venous thromboembolism in non-small cell lung cancer patients through data-independent acquisition mass spectrometry. Front Oncol 2023, 13, 1079719. doi: 10.3389/fonc.2023.1079719.
15. Ten Cate, V.; Koeck, T.; Panova-Noeva, M.; Rapp, S.; Prochaska, J.H.; Lenz, M.; Schulz, A.; Eggebrecht, L.; Hermanns, M.I.; Heitmeier, S.; Krahn, T.; Laux, V.; Münzel, T.; Leineweber, K.; Konstantinides, S.V.; Wild, P.S.; Collaborators: Investigators FOCUS BioSeq Study and VTEval Study. A prospective cohort study to identify and evaluate endotypes of venous thromboembolism: Rationale and design of the Genotyping and Molecular Phenotyping in Venous ThromboEmbolism project (GMP-VTE). Thromb Res 2019, 181, 84-91. doi: 10.1016/j.thromres.2019.07.019.
16. Jensen, S.B.; Latysheva, N.; Hindberg, K.; Ueland, T. Plasma lipopolysaccharide-binding protein is a biomarker for future venous thromboembolism: Results from discovery and validation studies. J Intern Med 2022, 292(3), 523-535. doi:10.1111/joim.13502.
17. Granholm, F.; Bylund, D.; Shevchenko, G.; Lind, S.B.; Henriksson, A.E. A Feasibility Study on the Identification of Potential Biomarkers in Pulmonary Embolism Using Proteomic Analysis. Clin Appl Thromb Hemost 2022, 28, 10760296221074347.doi: 10.1177/10760296221074347.
18. Gade, I.L.; Schultz, J.G.; Cehofski, L.J.; Kjærgaard, B.; Severinsen, M.T.; Rasmussen, B.S.; Vorum, H.; Honoré, B.; Kristensen, S.R. Exhaled breath condensate in acute pulmonary embolism; a porcine study of effect of condensing temperature and feasibility of protein analysis by mass spectrometry. J Breath Res 2021, 15(2). doi:10.1088/1752-7163/abd3f2.
19. Gade, I.L.; Schultz, J.G.; Brøndum, R.F.; Kjærgaard, B.; Nielsen-Kudsk, J.E.; Andersen, A.; Kristensen, S.R.; Honoré, B. Putative Biomarkers for Acute Pulmonary Embolism in Exhaled Breath Condensate. J Clin Med 2021, 10(21), 5165. doi:10.3390/jcm10215165.

20. Gade, I.L.; Riddersholm, S.J.; Stilling-Vinther, T.; Brøndum, R.F.; Bennike, T.B.; Honoré, B. A clinical proteomics study of exhaled breath condensate and biomarkers for pulmonary embolism. J Breath Res 2023, 18(1). doi: 10.1088/1752-7163/ad0aaa.
21. Iglesias, M.J.; Sanchez-Rivera, L.; Ibrahim-Kosta, M.; Naudin, C.; Munsch, G.; Goumidi, L.; Farm, M.; Smith, P.M.; Thibord, F.; Kral-Pointner, J.B.; Hong, M.G.; Suchon, P.; Germain, M.; Schrottmaier, W.; Dusart, P.; Boland, A.; Kotol, D.; Edfors, F.; Koprulu, M.; Pietzner, M.; Langenberg, C.; Damrauer, S.M.; Johnson, A.D.; Klarin, D.M.; Smith, N.L.; Smadja, D.M.; Holmström, M.; Magnusson, M.; Silveira, A.; Uhlén, M.; Renné, T.; Martinez-Perez, A.; Emmerich, J.; Deleuze, J.F.; Antovic, J.; Soria Fernandez, J.M.; Assinger, A.; Schwenk, J.M.; Souto Andres, J.C.; Morange, P.E.; Butler, L.M.; Trégouët, D.A.; Odeberg, J. Elevated plasma complement factor H related 5 protein is associated with venous thromboembolism. Nat Commun 2023, 14(1), 3280. doi: 10.1038/s41467-023-38383-y. Erratum in: Nat Commun 2023, 14(1), 7752. doi: 10.1038/s41467-023-43764-4.
22. Ten Cate V, Prochaska JH, Schulz A, Koeck T, Pallares Robles A, Lenz M, Eggebrecht L, Rapp S, Panova-Noeva M, Ghofrani HA, Meyer FJ, Espinola-Klein C,Lackner KJ, Michal M, Schuster AK, Strauch K, Zink AM, Laux V, Heitmeier S,Konstantinides SV, Münzel T, Andrade-Navarro MA, Leineweber K, Wild PS. Protein expression profiling suggests relevance of noncanonical pathways in isolated pulmonary embolism. Blood. 2021 May 13;137(19):2681-2693. doi:10.1182/blood.2019004571.
23. Jensen, S.B.; Hindberg, K.; Solomon, T.; Smith, E.N.; Lapek, J.D. Jr.; Gonzalez, D.J.; Latysheva, N.; Frazer, K.A.; Braekkan, S.K.; Hansen, J.B. Discovery of novel plasma biomarkers for future incident venous thromboembolism by untargeted synchronous precursor selection mass spectrometry proteomics. J Thromb Haemost. 2018, 16(9), 1763-1774. doi: 10.1111/jth.14220.
24. Bingqing, H.; Chuanbao, L.; Hexin, L.; Ying, Li.; Xuanmei, L.; Ye, L,; Junhua, Z.; Zhu, Z.; Xiaobo, Yu.; Zhenguo, Z.; Xiaomao, X.; Fei, X. Discovery of plasma biomarkers with data-independent acquisition mass spectrometry and antibody microarray for diagnosis and risk stratification of pulmonary embolism. Journal of Thrombosis and Haemostasis 19(7), 2021, 1738-1751. https://doi.org/10.1111/jth.15324.
25. Kong, Y., Tang, W., Kang, H. et al. Linear and non-linear proteome-wide association studies provide novel insight into venous thromboembolism. Nat Commun 16, 6517 (2025).
26. Smit, E.R.; Kreft, I.C.; Camilleri, E.; Burggraaf-van Delft, J.L.I.; van Rein, N.; van Vlijmen, B.J.M.; Hulshof, A.M.; van Bussel, B.C.T.; van Rosmalen, F.; van der Zwaan, C.; van de Berg, T.; Henskens, Y.; Ten Cate, H.; Coutinho, J.M.; Kruip, M.J.H.A.; Eikenboom, J.J.C.; Hoogendijk, A.J.; Cannegieter, S.C.; van den Biggelaar, M.; in collaboration with BEAT-COVID group and Dutch COVID & Thrombosis Coalition. Exploration of the plasma proteomic profile of patients at risk of thromboembolic events. Res Pract ThrombHaemost 2025, 9(2), 102713. doi: 10.1016/j.rpth.2025.102713
27. Ten Cate, V.; Koeck, T.; Prochaska, J.; Schulz, A.; Panova-Noeva, M.; Rapp, S.; Eggebrecht, L.; Lenz, M.; Glunz, J.; Sauer, M.; Ewert, R.; Halank, M.; Münzel, T.; Heitmeier, S.; Andrade-Navarro, M.A.; Lackner, K.J.; Konstantinides, S.V.; Leineweber, K.; Wild, P.S. A targeted proteomics investigation of the obesity paradox in venous thromboembolism. Blood Adv 2021, 5(14), 2909-2918. doi:10.1182/bloodadvances.2020003800.

28. von Zur Mühlen, C.; Koeck, T.; Schiffer, E.; Sackmann, C.; Zürbig, P.; Hilgendorf, I.; Reinöhl, J.; Rivera, J.; Zirlik, A.; Hehrlein, C.; Mischak, H.; Bode, C.; Peter, K. Urine proteome analysis as a discovery tool in patients with deep vein thrombosis and pulmonary embolism. Proteomics Clin Appl 2016, 10(5), 574-84. doi:10.1002/prca.201500105
29. Li, S.Q.; Qi, H.W.; Wu, C.G.; Zhang, X.J.; Yang, S.G.; Zhao, X.; Wu, Z.; Wang, Y.; Que, H.P.; Liu, S.J. Comparative proteomic study of acute pulmonary embolism in a rat model. Proteomics 2007, 7(13), 2287-99. doi: 10.1002/pmic.200500665.
30. Pallares Robles, A.; Ten Cate, V.; Lenz, M.; Schulz, A.; Prochaska, J.H.; Rapp, S.; Koeck, T.; Leineweber, K.; Heitmeier, S.; Opitz, C.F.; Held, M.; Espinola-Klein, C.; Lackner, K.J.; Münzel, T.; Konstantinides, S.V.; Ten Cate-Hoek, A.; Ten Cate, H.; Wild, P.S. Unsupervised clustering of venous thromboembolism patients by clinical features at presentation identifies novel endotypes that improve prognostic stratification. Thromb Res 2023, 227,71-81. doi: 10.1016/j.thromres.2023.04.023.

Non-proteomic studies included

1. Zagorski, J.; Marchick, M.R.; Kline, J.A. Rapid clearance of circulating haptoglobin from plasma during acute pulmonary embolism in rats results in HMOX1 up-regulation in peripheral blood leukocytes. J Thromb Haemost 2010,8(2),389-96. doi: 10.1111/j.1538-7836.2009.03704.x.
2. Yang, H.; Zhang, J.; Huan, Y.; Xu, Y.; Guo, R. Pentraxin-3 Levels Relate to the Wells Score and Prognosis in Patients with Acute Pulmonary Embolism. Dis Markers 2019,2019,2324515. doi: 10.1155/2019/2324515.
3. Ząbczyk, M.; Natorska, J.; Undas, A. Factor XIII and Fibrin Clot Properties in Acute Venous Thromboembolism. Int J Mol Sci 2021, 22(4), 1607. doi:10.3390/ijms22041607.
4. Bhatraju, P.K.; Morrell, E.D.; Stanaway, I.B.; Sathe, N.A.; Srivastava, A.; Postelnicu, R.; Green, R.; Andrews, A.; Gonzalez, M.; Kratochvil, C.J.; Kumar, V.K.; Hsiang, T.Y.; Gale, M. Jr.; Anesi, G.L.; Wyles, D.; Broadhurst, M.J.; Brett-Major, D.; Mukherjee, V.; Sevransky, J.E.; Landsittel, D.; Hung, C.; Altemeier, W.A.; Gharib, S.A.; Uyeki, T.M.; Cobb, J.P.; Liebler, J.M.; Crosslin, D.R.; Jarvik, G.P.; Segal, L.N.; Evans, L.; Mikacenic, C.; Wurfel, M.M. Angiopoietin-Like4 Is a Novel Marker of COVID-19 Severity. Crit Care Explor 2022, 5(1), e0827. doi: 10.1097/CCE.0000000000000827.

Table S2. Relevant proteins identified as biomarkers in venous thromboembolism.

Protein	Clinical Role	Biological Sample	Pathophysiological Relevance	Key Studies
Haptoglobin (Hp)	Diagnosis of VTE and PE; Prognosis (PE severity)	Serum / Plasma / Thrombus (Human, animal models)	An acute-phase protein that binds free hemoglobin, limiting oxidative endothelial injury; decreased levels reflect hemolysis associated with severe PE and pulmonary hypertension	[8,14,15,32]
Fibronectin (Fn)	Diagnosis of VTE and PE; clot characterization	Serum / Plasma fibrin clots (Human, animal models)	Extracellular matrix and coagulation protein that promotes clot stabilization, platelet adhesion, and resistance to fibrinolysis	[8,21,23]
Pentraxin-3 (PTX3)	Prognosis of PE (severity, mortality, recurrence)	Plasma (Human)	Marker of vascular and endothelial inflammation, reflecting local thromboinflammatory activation	[17]
Complement factor H–related protein 5 (CFHR5)	Diagnosis and recurrence risk of VTE	Plasma (Human)	Regulator of the alternative complement pathway; promotes platelet activation and thromboinflammation	[18]
Retinol-binding protein 4 (RBP4)	Acute PE diagnosis	Serum (Human)	Transport protein with pro-inflammatory effects mediated through TLR4 signaling	[15]

Transthyretin (TTR)	Prediction of future incidental VTE	Plasma / Serum (Human, animal models)	Transport protein involved in oxidative stress regulation and metabolic homeostasis	[8,44]
von Willebrand factor (vWF)	Diagnosis of VTE and PE; clot composition	Plasma / Fibrin clots (Human)	Mediator of platelet adhesion and endothelial dysfunction in thrombus formation	[21,23,27]
Coagulation factors (IX, V, VIII, XI, XII, XIII, fibrinogen, prothrombin)	Diagnosis and characterization of VTE	Plasma / Fibrin clots (Human)	Key components of the coagulation cascade influencing thrombus density, stability, and fibrinolytic resistance	[21,23,26]
α2-antiplasmin	PE diagnosis (reduced levels in acute phase)	Plasma fibrin clots (Human)	Inhibitor of plasmin; reduced levels contribute to altered clot lysis properties	[21,23]
Histones H3/H4 (NETs)	Specific marker of acute PE	Plasma fibrin clots (Human)	Components of neutrophil extracellular traps that enhance clot stability and propagate thromboinflammation	[23]
P-selectin	Severity and diagnosis of VTE in COVID-19	Plasma (Human)	Platelet and endothelial activation marker mediating leukocyte–platelet interactions	[27]
ANGPTL4	Diagnosis and prognosis of VTE in COVID-19	Plasma (Human)	Hypoxia-induced endothelial protein affecting vascular	[30]

			permeability and thrombosis	
Lipopolysaccharide-binding protein (LBP)	Diagnosis of PE; risk of DVT in women	Plasma (Human)	Inflammatory protein linking innate immunity with coagulation activation	[23,32,37]
Serum amyloid A-1 (SAA1), S100A8, Tenascin-C (TNC)	Prognosis of high-risk PE; cancer-associated VTE	Plasma (Human)	Markers of acute inflammation, neutrophil activation, and vascular remodeling	[32,45]
